# Draft Genome Sequences of Two Cystic Fibrosis Strains of Stenotrophomonas maltophilia, AU30115 and AU32848

**DOI:** 10.1128/MRA.01137-18

**Published:** 2018-09-20

**Authors:** Korin Eckstrom, Graham G. Willsey, John J. LiPuma, Matthew J. Wargo

**Affiliations:** a Department of Microbiology and Molecular Genetics, University of Vermont Larner College of Medicine, Burlington, Vermont, USA; b Department of Pediatrics and Communicable Disease, University of Michigan Medical School, Ann Arbor, Michigan, USA; Loyola University Chicago

## Abstract

Stenotrophomonas maltophilia is an opportunistic pathogen causing airway infection in people with cystic fibrosis (CF). Here, we report the draft genome sequences of two S. maltophilia strains, AU30115 and AU32848, recovered from CF patients.

## ANNOUNCEMENT

Stenotrophomonas maltophilia infects between 3% and 15% of cystic fibrosis (CF) patients, and based on recent epidemiologic assessments, it is increasing in prevalence in this population (1–3). Infection of airways by S. maltophilia has been linked to poor outcomes, including decreased time to exacerbation, increased risk of death or transplant, CF-related diabetes, and decreased lung function (1–14). While S. maltophilia infection has not been proven to be causative for these outcomes, it can promote lung inflammation (15, 16) and is inherently multidrug resistant (9, 17). Despite its prevalence and clinical correlations, our understanding of S. maltophilia virulence and response to host defenses lags far behind that of other CF pathogens. Here, we report the draft genome sequences of two clinical strains of S. maltophilia. S. maltophilia AU30115 was recovered from an adult CF patient in Texas in 2014, and S. maltophilia AU32848 was recovered from an adult CF patient in Nebraska in 2015. Genomic DNA was purified from LB-grown cultures via cetyltrimethylammonium bromide (CTAB) extraction (18), assessed for integrity with a Bioanalyzer, and quantified using a Qubit fluorometer.

The genome sequencing of strains AU30115 and AU32848 was performed by the Vermont Integrative Genomics Resource (VIGR). Two separate runs of an Illumina HiSeq 1500 system were performed using Nextera library preparation, a 150-bp single end and an 85-bp single end in rapid run mode. Sequences were trimmed of adaptors and low-quality bases (<Q30) using Trim Galore v. 0.5.0 (http://www.bioinformatics.babraham.ac.uk/projects/trim_galore/), and quality was assessed using FastQC v. 0.11.6 (http://www.bioinformatics.babraham.ac.uk/projects/fastqc) prior to assembly. Trimmed reads from each run were combined into a single file per isolate containing 1,592,793 and 1,813,244 reads in AU30115 and AU32848, respectively. Assembly was performed using Unicycler v. 0.4.6 (19) with default parameters, including read error correction and assembly polishing. Assembly graphs were visualized and compared to the reference sequence using Bandage (20). Contigs with no match to the reference were queried against the NCBI nonredundant (nr) database and removed if they were found to be the result of eukaryotic or reagent contamination. Contigs shorter than 200 bp were also removed. After these removals, *N*_50_ values of 94,013 and 63,013 bp were obtained for AU30115 and AU32848, respectively. Closest genomic neighbors and nucleotide identities were determined using the Microbial Genomes Atlas (MiGA) (21) to query available genomes in the NCBI prokaryotic genome database.

The assembly of AU30115 resulted in 89 contigs at 41× coverage (total bases/genome size) with a total genome size of 4,628,959 bp and a GC content of 66.42%. Coverage was calculated as total bases/genome size for each isolate using readfq (https://github.com/billzt/readfq). Assembly of AU32848 resulted in 155 contigs at 42× coverage, a total genome size of 4,383,122 bp, and a GC content of 66.64%. The closest genome for both strains was the reference strain S. maltophilia K279A NCTC 10943, with average nucleotide identity (ANI) values of 99.76% and 98.3% for AU30115 and AU32848, respectively.

Annotation was performed using the NCBI Prokaryotic Genome Annotation Pipeline (PGAP) (22). The assembled genome of AU30115 contains 4,229 coding DNA sequences (CDS), 4,134 coding genes, 3 complete rRNA operons, and 66 tRNAs. The genome of AU32848 contains 3,990 CDS, 3,930 coding genes, 2 rRNA operons, and 48 tRNAs.

### Data availability.

This whole-genome shotgun project has been deposited in GenBank under the accession no. GCF_003385015 and GCA_003385135, BioProject no. PRJNA483996, and SRA study no. SRP158885. The versions described in this paper are the first versions, GCA_003385015.1 and GCA_003385135.1.
